# Medical Topography of Chester, by Dr. Pigot

**Published:** 1813-05

**Authors:** John M. B. Pigot

**Affiliations:** Nicholas-street, Chester


*To the Editors of the Medical and Physical Journal.*

GENTLEMEN,

IN your Number for this month, you request your correspondents will furnish lists of medical practitioners, and of medical institutions, in their neighbourhood. As the utility of such record seems evident, I hasten to furnish the materials for Chester, confining myself solely to the city, the medical residents of which are as follow:

PHYSICIANS.

William Currie, M.D. of the University of Edinburgh.
*William M. Thackeray, M.D. of Cambridge.
Robert F. Currie, M.D. of Edinburgh.
*John M. B. Pigot, M.D. of Edinburgh.

SURGEONS AND APOTHECARIES, ALL PRACTISING MIDWIFERY.

| | |
|---|---|
| Daniel Orred, | *Samuel N. Bennett, |
| *Griffiths Rowland, | *Thomas Bagnall, |
| James Okell, | John Davies, |
| George N. Hill, | Joseph Bromfield, |
| John Harrison, | *Owen Titley, |
| George Harrison, | Edw. Simon, Inf. House Apo. |

Connected with the Infirmary are extensive fever-wards for male and female patients, open to any one applying, on the payment of a small weekly stipend.

There is a charitable society of ladies, for the delivery of married women at their own houses by a midwife, assisted by the surgeons of the city in rotation, if required.

There is a society for the purchase and circulation of medical journals and books among its own members, of which a physician and surgeon, alternately, is president; Dr. Pigot, the secretary.

There are three damp and inadequate cells in the poorhouse for the reception of lunatics; and, as far as I know,

* Medical and Surgical Officers to the County Infirmary.

there there is not any other receptacle for maniacs throughout Cheshire and North Wales.

Although I might be able to furnish lists of the practitioners of the neighbouring market-towns, I feel myself unable to give an accurate general list of the county, and prefer leaving it to other hands, rather than offer dubious intelligence. I remain, Gentlemen,

Your most obedient Servant,

*Nicholas-street, Chester,* JOHN M. B. PIGOT, M.D.

*March* 9, 1813.

